# Identification of Species of Nontuberculous Mycobacteria Clinical Isolates from 8 Provinces of China

**DOI:** 10.1155/2016/2153910

**Published:** 2016-11-02

**Authors:** Haican Liu, Lulu Lian, Yi Jiang, Mingxiang Huang, Yunhong Tan, Xiuqin Zhao, Jingrui Zhang, Qin Yu, Jiao Liu, Haiyan Dong, Bing Lu, Yimou Wu, Kanglin Wan

**Affiliations:** ^1^State Key Laboratory for Infectious Disease Prevention and Control, Collaborative Innovation Center for Diagnosis and Treatment of Infectious Diseases, National Institute for Communicable Disease Control and Prevention, Chinese Center for Disease Control and Prevention, Beijing 102206, China; ^2^Pathogenic Biology Institute, University of South China, Hengyang, Hunan 421001, China; ^3^Fuzhou Pulmonary Hospital (Clinical Teaching Hospital of Fujian Medical University), Fuzhou, Fujian 350008, China; ^4^Hunan Institute for Tuberculosis Control/Hunan Chest Hospital, Changsha, Hunan 410013, China

## Abstract

Pulmonary diseases caused by nontuberculous* mycobacteria* (NTM) are increasing in incidence and prevalence worldwide. In this study, we identified NTM species of the clinical isolates from 8 provinces in China, in order to preliminarily provide some basic scientific data in the different species and distribution of NTM related to pulmonary disease in China. A total of 523 clinical isolates from patients with tuberculosis (TB) diagnosed clinically from 2005 to 2012 were identified to the species using conventional and molecular methods, including multilocus PCR,* rpoB* and* hsp65* PCR-PRA,* hsp65*,* rpoB*, and* 16S*-*23S* internal transcribed spacer region sequencing. The isolates were identified into 3 bacterium genera, including NTM,* Gordonia bronchialis,* and* Nocardia farcinica*, and, for the 488 NTM isolates, 27 species were identified. For all the 27 species of NTM which were found to cause pulmonary infections in humans, the most prevalent species was* M. intracellulare*, followed by* M. avium* and* M. abscessus*. And seven other species were for the first time identified in patients with TB in China. NTM species identification is very important for distinguishing between tuberculosis and NTM pulmonary diseases, and the species diversity drives the creation of diverse and integrated identification methods with higher accuracy and efficacy.

## 1. Introduction

The* Mycobacterium* genus consists of species belonging to the* Mycobacterium tuberculosis* complex (MTBC),* Mycobacterium leprae,* and nontuberculous mycobacteria (NTM). NTM are environmental opportunistic pathogens in some cases [1, 2] and nonpathogenic in others [1, 3]. To date, more than 160 NTM species have been recognized, while many others await classification [4]; approximately one-third have been associated with diseases in humans [4]. The importance of NTM has recently increased and the pathogenic roles of many of these mycobacteria have been clearly defined [5]. Known reasons for this include (1) raising awareness, gaining more and more interests for these genera; (2) increased number of immunosuppressed patients including HIV; (3) increased number of patients with preexisting lung disease; and (4) mostly increased distribution of methods to detect NTM species [6, 7].

Tuberculosis (TB) remains a major public health problem in China. Identification of NTM has been conducted only in a few clinical laboratories in China, so misdiagnosis and incorrect treatment are commonly given to the patients infected with NTM. As some of the NTM species are natively insensitive to the commonly used antituberculosis drugs, the NTM infected patients may be treated as drug-resistant TB, which result in some of the TB patients getting misdiagnosis and inappropriate therapy [8]. Once the anti-TB therapy fails, the treatment of NTM infection might be delayed several months or even more. That is why now increased awareness of NTM identification is important. Distinguishing the diseases caused by different pathogens to avoid misdiagnosis allows for accurate treatment. American Thoracic Society documents state that NTM should generally be identified to the species level [5]. In the current study, we collected 523 clinical NTM isolates from 8 provinces in China and identified them to species or subspecies level. These data may preliminarily provide an insight into the different species and distribution of NTM related to pulmonary disease in China and help to prevent treatment failure by underscoring the importance of differentiating mycobacterial species.

## 2. Materials and Methods

### 2.1. Ethics Statement

This study was approved by the Ethics Committee of the National Institute for Communicable Disease Control and Prevention, Chinese Center for Disease Control and Prevention. All participants provided written informed consent.

### 2.2. Mycobacterial Isolates

**Figure 1:**
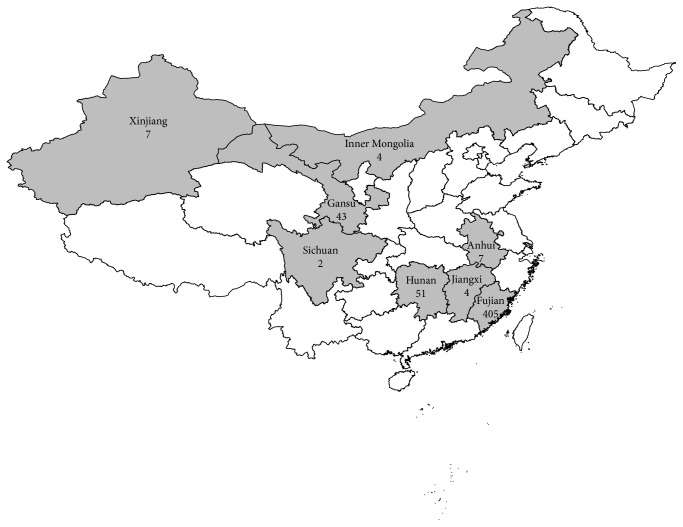
Distribution of clinical isolate strains in the 8 provinces. There are different colors in the different regions. The numbers of isolates and the constituent ratios were marked under the corresponding provinces.

From 2005 to 2012, 523 clinical isolates were provided by the province's TB hospital or center for TB control in 8 provinces or autonomous regions, which include Anhui, Fujian, Gansu, Hunan, Jiangxi, Inner Mongolia, Sichuan, and Xinjiang, and conserved at the National Institute for Communicable Disease Control and Prevention, Chinese Center for Disease Control and Prevention, Beijing, China, for NTM species identification.  Table 1 and Figure 1 show the distribution of these clinical strains in detail. All of the isolates, isolated from sputum of TB patients, were primarily identified as NTM by each province's TB hospital laboratory using conventional biochemical testing with* p*-nitrobenzoic acid/2-thiophene carboxylic acid hydrazide (PNB/TCH) media and incubated at 37°C for 3~4 weeks following a standard protocol [9, 10].

### 2.3. DNA Sample Preparation

For the DNA extraction, a loopful of organisms grown on Löwenstein-Jensen (L-J) medium incubated at 37°C for 3-4 weeks was suspended in 200 *μ*L of pH 8.0 TE buffer (10 mM Tris-HCl; 1 mM ethylenediaminetetraacetic acid). The bacterial cells were lysed with lysozyme and the genomic DNA was isolated using cetyltrimethylammonium bromide [11, 12].

### 2.4. Multilocus PCR

According to Huard et al. [13], the MTBC PCR typing panel using 7 primer pairs amplifying within the loci* 16S rRNA*,* Rv0577*,* IS1561*′,* Rv1510*,* Rv1970*,* Rv3877/8*, and* Rv3120* results in pattern of amplification products from all of the reactions that allow differentiation of NTM from MTBC subspecies by agarose gel electrophoresis. PCR amplifications were performed using a program with an initial denaturation step of 5 min at 94°C followed by 35 cycles of 1 min at 94°C, 1 min at 60°C, and 1 min at 72°C and ending with a final elongation step of 10 min at 72°C. The PCR products and a 100 bp ladder (Beijing CoWin Biotech Co., Ltd., Beijing, China) were visualized using 2% agarose gel electrophoresis and ethidium bromide (EB) staining.

### 2.5. Amplification of* rpoB* and* hsp65* Partial DNA

A specific region of the* rpoB* gene was amplified using primers* rpoB *F (5′-TCAAGGAGAAGCGCTACGA-3′) and* rpoB *R (5′-ATGTTGATCAGGGTCTGC-3′), resulting in a 360 bp PCR product [14, 15]. Primers Tbll (5′-ACCAACGATGGTGTGTCCAT-3′) and Tb12 (5′-CTTGTCGAACCGCATACCCT-3′) amplified a 441 bp fragment between positions 398 and 836 of the published* hsp65* gene [16, 17]. The composition of the PCR mixture (50 *μ*L) was 25 *μ*L of 2x Taq MasterMix (Beijing CoWin Biotech Co., Ltd.), 2 *μ*L of each primer at 10 *μ*M, 5 *μ*L of DNA-containing supernatant, and 16 *μ*L of ddH_2_O. The reaction was subjected to 35 cycles of amplification (1 min at 94°C, 1 min at 60°C, and 1 min at 72°C), followed by 10 min of extension at 72°C. The PCR products and a 100 bp ladder (Beijing CoWin Biotech Co., Ltd.) were visualized using 2% agarose gel electrophoresis and EB staining. The PCR products were purified using a Quick DNA Purification Kit (Beijing CoWin Biotech Co., Ltd.).

### 2.6. PRA-*hsp65 *[17] and PRA-*rpoB *[15] Methods and Analysis

The purified PCR products were digested with restriction enzyme (Takara Biotechnology [Dalian] Co., Ltd.). The* hsp65 *gene PCR products were digested with* Bst*P I (1 *μ*L of* Bst*P I, 15 *μ*L of DNA, 2 *μ*L of 10x H buffer, and 2 *μ*L of ddH_2_O at 60°C for 2 h) and* Hae *III (1 *μ*L of* Hae *III, 15 *μ*L of DNA, 2 *μ*L of 10x M buffer, and 2 *μ*L of ddH_2_O at 37°C for 2 h). For* rpoB* gene's PCR products, the reactions contained* Msp *I (1 *μ*L of* Msp *I, 15 *μ*L of DNA, 2 *μ*L of 10x T buffer, and 2 *μ*L of 0.1% BSA) and* Hae* III (1 *μ*L of* Hae *III, 15 *μ*L of DNA, 2 *μ*L of 10x M buffer, and 2 *μ*L of ddH_2_O) and both were incubated at 37°C for 2 h. The resulting restriction fragments were separated using electrophoresis in a 4% agarose gel (MetaPhor® Agarose, LONZA) with a 20 bp ladder (Takara Biotechnology [Dalian] Co., Ltd.) as molecular standard. The gels were stained with EB and then photographed using a ChemiDoc™ XRS+ System (Bio-Rad Laboratories, Inc., Hercules, USA). The restriction fragment sizes were estimated using Image Lab software (Bio-Rad Laboratories, Inc., Hercules, USA). Species identification was confirmed according to restriction fragment size [16–25].

### 2.7. PCR Sequencing and Multisequence Alignment

When correct identification by PRA was impossible or discordant results were obtained between PRA and phenotypic methods, gene sequencing was performed to properly identify the mycobacterial species [26]. To identify the isolates to the species level, we firstly sequenced partial* hsp65* sequences of the strains that were not identified by PRA and then for the other strains <97% matched of the existing* hsp65* sequences, also sequencing the partial* rpoB *gene and the 16S–23S internal transcribed spacer (ITS) region, using the previously published primers and methods [19, 27, 28]. The PCR products were sequenced by Beijing Tsingke Bio Tech Co., Ltd. (Beijing, China). The obtained sequences were compared with those in the GenBank (National Center for Biotechnology Information: http://www.ncbi.nlm.nih.gov/) DNA sequence database, and species identification was confirmed if a 97% match was achieved [17, 29].

### 2.8. Statistical Analysis

Data are presented as the frequencies for NTM species in 8 provinces. The *χ* ^2^ test was used to compare differences in proportions; *P* < 0.05 was considered statistically significant. All statistical analyses were performed using statistical software (SPSS 16.0; SPSS; Chicago, IL).

## 3. Results

**Figure 2:**
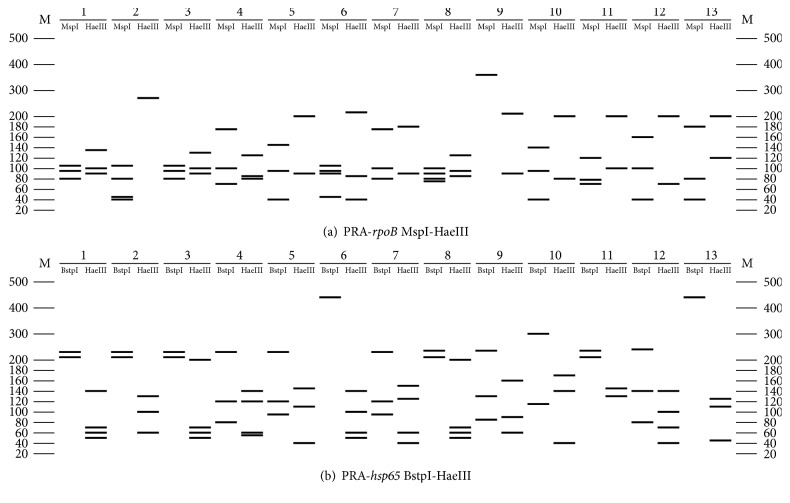
The PRA-*hsp65* gene and PRA-*rpoB* gene patterns of 13 different species of NTM in our study. M: 20 bp DNA ladder; 1:* M. abscessus*; 2:* M. avium*; 3:* M. chelonae*; 4:* M. fortuitum*; 5:* M. gordonae*; 6:* M. holsaticum*; 7:* M. intracellulare*; 8: formerly* M. massiliense*; 9:* M. monacense*; 10:* M. neoaurum*; 11:* M. seoulense*; 12:* M. shimoidei*; and 13:* M. szulgai*.

**Figure 3:**
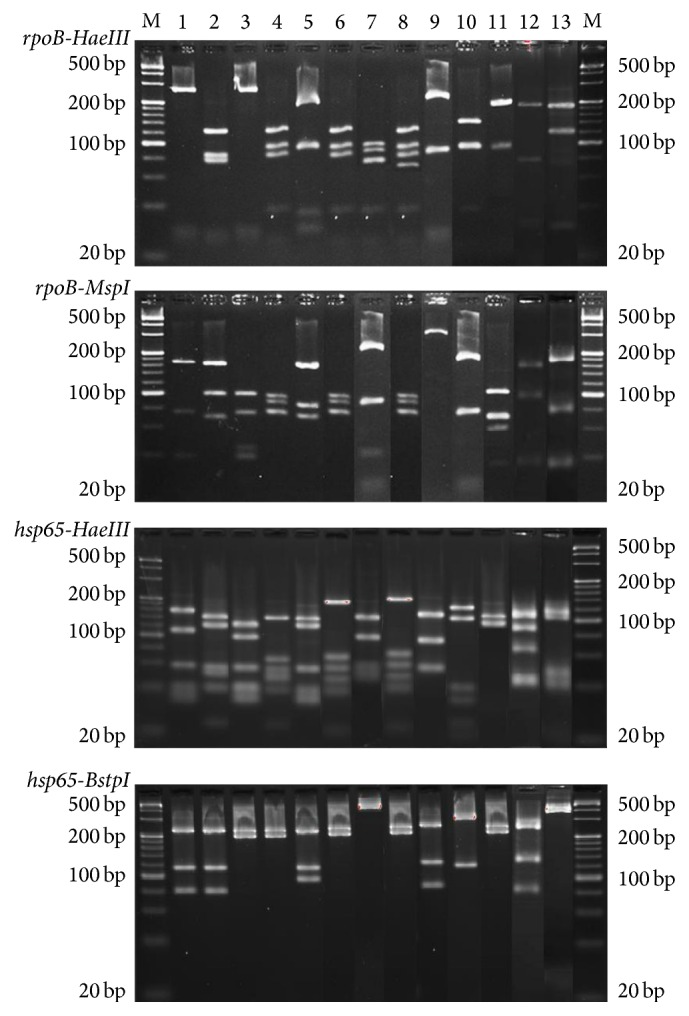
The results on gel electrophoresis of different species of NTM with PRA-*hsp65* and PRA-*rpoB*. M: 20 bp DNA ladder; 1: HN11051,* M. gordonae*; 2: HN11052,* M. fortuitum*; 3: HN1053,* M. avium*; 4: HN11054,* M. abscessus*; 5: FJ05232,* M. intracellulare*; 6: FJ10024,* M. chelonae*; 7: FJ10038,* M. holsaticum*; 8: FJ10101, formerly* M. massiliense*; 9: FJ10130,* M. monacense*; 10: HN11058,* M. neoaurum*; 11: FJ10157,* M. seoulense*; 12: FJ12019,* M. shimoidei*; and 13: FJ12159,* M. szulgai*.

**Figure 4:**
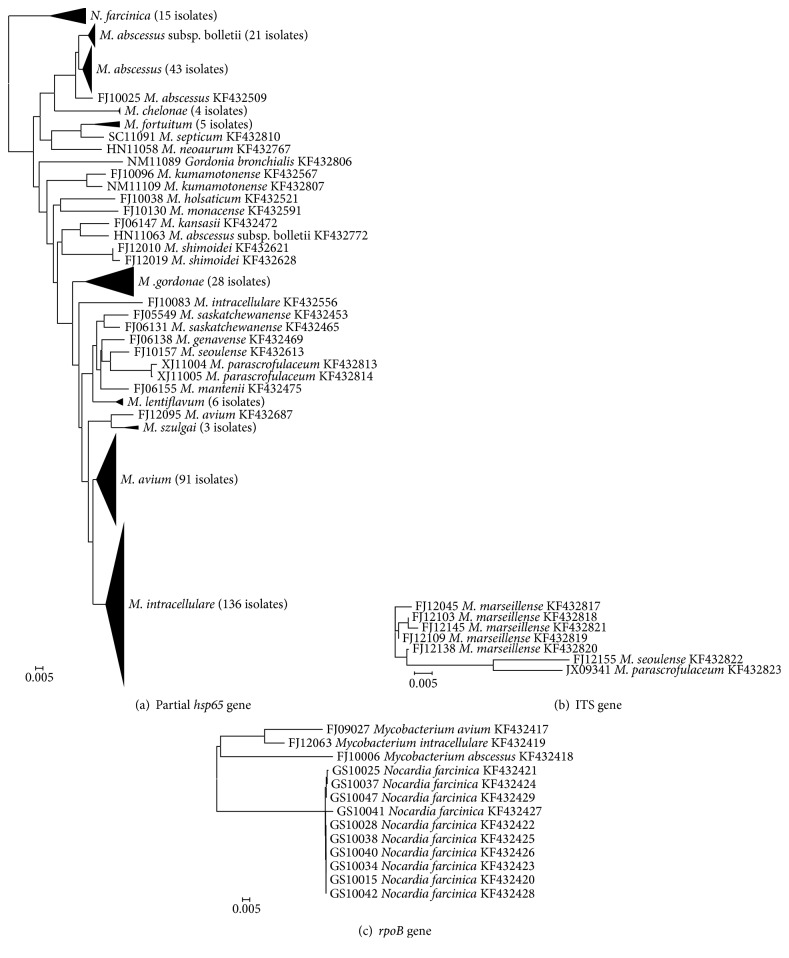
The phylogenetic trees (N-J) constructed by the gene sequences.

A total of 523 clinical isolates primarily identified using L-J and PNB/TCH tests at the local province's TB hospital laboratories of 8 provinces in China were confirmed to be NTM with the same results of PNB/TCH tests and Multilocus PCR. And according to the results of PRA-*hsp65* and PRA-*rpoB* screening, it was shown that, using the 2 gene PRA patterns (Figure 2), 302 (57.74%; 302/523) clinical isolates were identified to 13 species of NTM, while the remaining 221 (42.26%; 221/523) isolates presented unknown restriction patterns or different patterns that were not comparable to those of the reference strains (Table 3).  Figure 3 shows the representative PRA-*hsp65* and PRA-*rpoB* patterns of 13 different species of NTM. Herein, the remaining 221 isolates were further analyzed by determination of the nucleotide sequence of the* hsp65, rpoB,* and* ITS* genes (the accession numbers of these sequences can be found in the Supplementary Material available online at http://dx.doi.org/10.1155/2016/2153910). A total of 88.69% (196/221) were identified to the species level according to the criterion of the first 97% distinct* hsp65* sequence match, while the other 25 (11.31%; 25/221) isolates were identified to the species level using* rpoB* and* ITS* sequencing (Figure 4).

In summary, we obtained 29 species (Table 2), including 27 NTM (488 strains), one* Gordonia bronchialis* (2 strains), and one* Nocardia farcinica* (32 strains), and for the 27 NTM species they were identified as belonging to the* Mycobacterium* genus (Table 2), including 6 slowly growing scotochromogenic species (Runyon Groups II), 11 slowly growing nonpigmented species (Runyon Groups III), and 10 rapidly growing species (Runyon Groups IV).

Table 2 also shows the frequency of the different species identified from the 523 isolates using molecular methods. As in Fujian and Hunan provinces,* M. intracellulare* and* M. avium* account for 56.60%, more than half of the total 523 isolates. In these 8 provinces, the proportion of* M. avium* and* M. intracellulare* (106 strains and 206 strains, resp.) was 59.66%. According to Table 1, there were 404 (12.87%) NTM in these 3139 strains in Fujian, followed by Hunan, and 1133* Mycobacterium* strains including 50 NTM (4.41%). Using SPSS 16.0 software package, we found that the difference between NTM and MTB species distribution in Hunan and Fujian province is statistically significant (*χ* ^2^ = 62.69, *P* = 0.00) according to 0.05 significance level. We may consider that the NTM infection rate of Fujian is higher than that of Hunan province.

## 4. Discussion

Recently the prevalence of NTM diseases in China is rising as the incidence of the pulmonary disease which was predisposed to NTM is increasing [30, 31], and the coinfection of HIV also contributed to this increase. The clinical symptoms and signs of NTM caused diseases are often hard to differentiate from those of MTBC-induced diseases, and the clinical NTM isolates are often of natural resistance to drugs that for TB infection treatment are linked to inadequate treatment in the public health system and the private hospital system, even in TB hospitals [32]. Also, the management and treatment of infected patients, in addition to the implemented epidemiological control methods, must reflect the encountered specific mycobacterial species [33]; therefore, it is important to accurately identify the NTM infections. Furthermore, Although there are diagnostic criteria of NTM diseases in the American Thoracic Society documents, there is not enough known about most other NTM species to ensure that these diagnostic criteria are universally applicable to all NTM respiratory pathogens [5]. So much more research of NTM infections should be performed.

It is well known that NTM are ubiquitous environmental microorganisms that can be found in a variety of ecosystems [34]. In the selected 8 provinces, most of isolates were collected in Fujian and Hunan province. Fujian lies in the southern coast of China, and the local subtropical climate in this area may affect the NTM recovery. We analyzed the difference between the species distribution in Hunan and Fujian province that is statistically significant (*χ* ^2^ = 62.69, *P* = 0.00) according to 0.05 significance level. We may consider that NTM incidence of Fujian province is more than that of Hunan. One hypothesis is that water exposure is important, also sea-water exposure [35]. And the fact that Fujian has a long sea border may potentially explain the significant differences found in the isolation frequency in comparison to that of the Hunan province since water exposure including sea water may be relevant for prevalence of different NTM strains. In the future, we will try to collect more* Mycobacterium* isolates form different provinces and to identify more NTM species, so that comparisons between the species distribution in different provinces could be made.

NTM can cause disseminated diseases in severely immunocompromised patients and affect organs such as the skin and soft tissues [5, 26]. These strains were isolated from sputum of TB patients; maybe these NTM can cause lung infections.* M. marseillense*,* M. saskatchewanense*,* M. seoulense*,* M. kumamotonense*,* M. stomatepiae*, and* M. mantenii* were first identified in patients with TB in China. More and more lung infection-causing NTM species are being found and need to be confirmed clinically. In contrast with MTBC members, NTM are free-living and ubiquitously distributed microorganisms with diverse abilities to cause disease in human beings and have emerged in the past few decades as being frequently associated with HIV coinfection [5]. NTM species are of great significance for control and prevention of TB.

Two other genera,* Nocardia *and* Gordonia*, were also identified in these isolates as well, but their findings were not differentiated using traditional identification methods such as acid-fast stains, PNB/TCH differential media, and multilocus PCR. Furthermore, although* N. farcinica* and* G. bronchialis* could be discriminated from the mycobacteria species using* hsp65* sequencing. The question is why* Nocardia *spp. are gram-positive, partially acid-fast, opportunistic pathogens.* G. bronchialis *is a slightly acid-fast organism that has been identified in both the sputum of patients with pulmonary disease and the soil [36]. As such, special and rapid methods should be used to identify partially or slightly acid-fast species. A total of 33* N. farcinica *and 2* G. bronchialis *strains were differentiated from the suspected NTM by PRA and sequencing, which may lead to greater diagnostic accuracy and a more effective treatment system. PRA and sequencing methods are accurate and rapid but costly, which creates a high burden for China, a developing country.

PRA-*hsp65* and PRA-*rpoB* are simple, rapid, and accurate to a certain extent and have been routinely used to identify mycobacteria. Here we used them to identify the obtained 300 clinical isolates. Because of the limitations of PRA and the unknown restriction pattern, the remaining 221 isolates were further analyzed using nucleotide sequencing of the* hsp65, rpoB*, and* ITS* genes. Due to* hsp65* gene hypervariability, sequence analysis of the Telenti fragment (441 bp) has become routine in taxonomical studies and in the identification of clinical* Mycobacterium *isolates [37]. The* rpoB* and* ITS* gene sequencing processes are auxiliary tools. Strain diversity results in the need for diverse identification methods and integrated solutions that create more accurate and effective results.

In this study we think there were limitations, for example, in the sampling of strains from some provinces. We collected clinical positive acid-fast strains that were isolated from sputum samples of clinically diagnosed TB patients in each province. But for some reasons, such as China being very big and different provinces' conditions, there were significant differences among the clinical isolates collected from different provinces (Table 1). Though the strain data had no representativeness of the samples in China, it still could preliminarily provide some basic scientific data in the different species and distribution of NTM related to pulmonary disease and some new NTM species associated to pulmonary disease. And, a lot of work still needs to be done in the future.

In summary, we identified 27 species of NTM, including* M. marseillense*,* M. saskatchewanense*,* M. seoulense*,* M. kumamotonense*,* M. stomatepiae*,* M. mantenii*, and* G. bronchialis*, that were first identified in patients with tuberculosis diagnosed clinically. And combining use of multilocus PCR, PRA-*hsp65*, and PRA-*rpoB* and genes sequencing methods can efficiently identify NTM isolates. This study can preliminarily provide some basic scientific data in the different species and distribution of NTM related to pulmonary disease in China, so it will be practical application and reference value for NTM pulmonary disease and tuberculosis control and cure.

## Supplementary Material

In the Supplementary Material, we supplied the detail Genbank accession number of all the sequences which were used to identify the genotype and to construct the N-J phylogeny trees of the sample isolates.

## Figures and Tables

**Table 1:** The distribution of clinical NTM strains throughout 8 Chinese provinces.

Province	*Mycobacterium* strain number	NTM number	Rate of NTM in *Mycobacterium* (%)
Anhui	180	7	3.9
Fujian	3139	405	12.9
Gansu	623	43	6.9
Hunan	1133	51	4.5
Jiangxi	93	4	4.3
Inner Mongolia	103	4	3.9
Sichuan	44	2	4.5
Xinjiang	140	7	5.0

Total	5455	523	9.6

NTM: nontuberculosis mycobacterium.

**Table 2:** Frequency of 29 different species identified by molecular methods from 523 isolates.

Genus (*n*)	Species	Anhui	Fujian	Gansu	Hunan	Jiangxi	Neimeng	Sichuan	Xinjiang	Overall *n* (%)
*Gordonia* (2)	*Gordonia bronchialis *		1				1			2 (0.38)
NTM (488)	*M. abscessus*		55		5					60 (12.30)
	*M. avium*	2	88		13	2		1		106 (21.72)
	*M. chelonae*		1	5						6 (1.23)
	*M. colombiense*		1						2	3 (0.61)
	*M. fortuitum*		9		2					11 (2.25)
	*M. genavense*		1							1 (0.20)
	*M. gordonae*		28	4	2					34 (6.97)
	*M. holsaticum *		1							1 (0.20)
	*M. intracellulare*	4	173	2	22		2		3	206 (42.21)
	*M. kansasii*		1		1					2 (0.41)
	*M. kumamotonense*		1				1			2 (0.41)
	*M. lentiflavum*		6							6 (1.23)
	*M. mantenii*		1							1 (0.20)
	*M. marseillense*		5							5 (1.02)
	Formerly* M. massiliense*	1	19		2					22 (4.51)
	*M. monacense*		1							1 (0.20)
	*M. neoaurum*				2					2 (0.41)
	*M. parascrofulaceum*					1			2	3 (0.61)
	*M. phocaicum*				1					1 (0.20)
	*M. saskatchewanense*		2							2 (0.41)
	*M. seoulense*		2							2 (0.41)
	*M. septicum*		1					1		2 (0.41)
	*M. setense*			1						1 (0.20)
	*M. shimoidei*		2							2 (0.41)
	*M. stomatepiae*		1							1 (0.20)
	*M. szulgai*		3							3 (0.61)
	*M. triplex*		2							2 (0.41)
*Nocardia* (32)	*N. farcinica*			31	1	1				33 (6.31)

NTM: nontuberculous mycobacteria.

**Table 3:** Fragment lengths (base pairs) of the isolates with the patterns using the PRA-*rpoB* and PRA-*hsp65 *methods.

Number	Species	PRA^d^-*rpoB*		PRA^d^-*hsp65*		Species (*n*)	Total
		*Msp*I	*Hae*III	*Bstp*I	*Hae*III		
1	*M. abscessus*	105, 95, 80	135, 100, 90	230, 210	140, 70, 60, 50	*M. abscessus* (44)	44
2	*M. avium*	105, 80, 45, 40	270	230, 210	130, 100, 60	*M. avium *(73)	73
3	*M. chelonae*	105, 95, 80	130, 100, 90	230, 210	200, 70, 60, 50	*M. chelonae *(1)	1
4	*M. fortuitum*	175, 100, 70	125, 85, 80	230, 120, 80	140, 120, 60, 55	*M. fortuitum *(8)	8
5	*M*. *gordonae* ^a^	145, 95, 40	200, 90	230, 120, 95	145, 110, 40	*M. gordonae *(2)	12
		145, 95, 40	210, 95, 90	235, 120, 85	160, 115, 60	*M. gordonae *(4)	
		175, 80, 40	270	230, 120, 80	160, 110, 60	*M. gordonae *(6)	
6	*M. holsaticum*	105, 95, 90, 45	215, 85, 40	440	140, 100, 60, 50	*M. holsaticum *(1)	1
7	*M*. *intracellulare* ^b^	175, 100, 80	180, 90	230, 120, 95	150, 125, 60, 40	*M. intracellulare *(105)	154
		180, 90, 80	185, 105	240, 120, 100	140, 125, 60, 40	*M. intracellulare *(2)	
		180, 90	180, 90, 80	240, 120, 100	140, 125, 60, 40	*M. intracellulare *(47)	
8	Formerly* M. massiliense*	100, 90, 80, 75	125, 95, 85	235, 210	200, 70, 60, 50	Formerly* M. massiliense *(4)	4
9	*M. monacense*	359	210, 90	235, 130, 85	160, 90, 60	*M. monacense *(1)	1
10	*M. neoaurum*	140, 95, 40	200, 80	300, 115	170, 140, 40	*M. neoaurum *(1)	1
11	*M. seoulense*	120, 78, 70	200, 100	235, 210	145, 130	*M. seoulense *(1)	1
12	*M. shimoidei*	160, 100, 40	200, 70	240, 140, 80	140, 100, 70, 40	*M. shimoidei *(1)	1
13	*M. szulgai*	180, 80, 40	200, 120	440	125, 110, 45	*M. szulgai *(1)	1
14	Not identified	—^c^	—^c^	—^c^	—^c^	—^c^	221

^a^Different types of *M. gordonae* showed different patterns that need more research in the future.

^b^ *M. intracellulare *displayed the different patterns in our study.

^c^Number of fragment lengths that are not identified.

^d^PRA: PCR-restriction fragment-length polymorphism analysis.

## Additional Points

*GenBank Sequence Accession Numbers.* The sequences used in the manuscript have been submitted to the GenBank sequence database recently. In the GenBank sequence database, the accession numbers of sequences are KF432417–KF432429 (13 partial* rpoB* genes), KF432430–KF432816 (387 partial* hsp65* genes), and KF432817–KF432823 (7 ITS sequences) (see Supplementary Material accession numbers).
